# Supplementary figures and images for: Longevity and mortality in cats: A single institution necropsy study of 3108 cases (1989–2019)
Source: PLoS One. 2022 Dec 29;17(12):e0278199. doi: 10.1371/journal.pone.0278199 (PMC9799304; doi:10.1371/journal.pone.0278199)

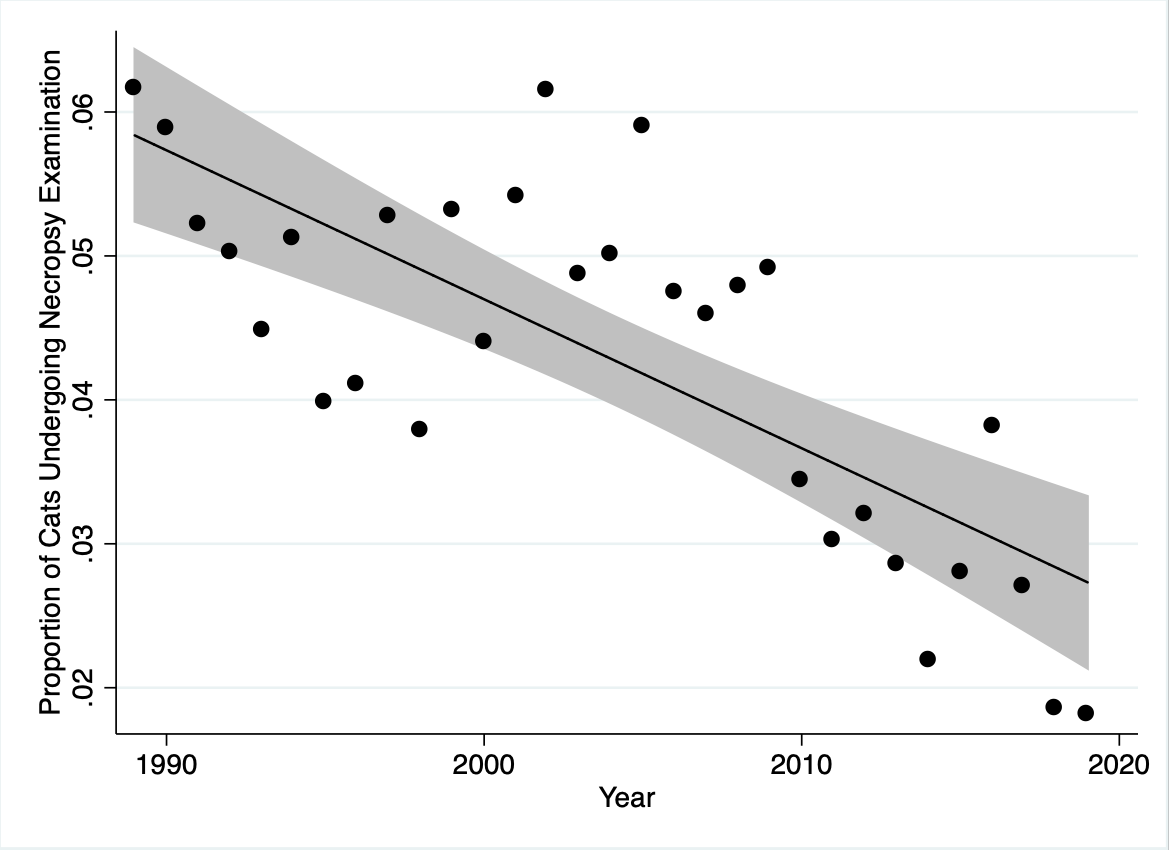

Supplement: S1 Fig — Graph showing the proportion of cats undergoing necropsy by year from 1989–2019. In addition, the regression line showing a decrease in the proportion of cats undergoing a necropsy along with the 95% confidence intervals represented by the shaded area is shown. (TIF) [file pone.0278199.s001.tif]
