# Supplementary material for: Longevity and mortality in cats: A single institution necropsy study of 3108 cases (1989–2019)
Source: PLoS One. 2022 Dec 29;17(12):e0278199. doi: 10.1371/journal.pone.0278199 (PMC9799304; doi:10.1371/journal.pone.0278199)
Supplement: S2 Table — (DOCX) [file pone.0278199.s003.docx]

Supplemental Table 2:

| **Cause of Death** | **Number** | **Percent** |
| --- | --- | --- |
| Amyloidosis | 12 | 0.39 |
| Anemia - Undetermined Cause | 26 | 0.84 |
| Anesthesia Related | 9 | 0.29 |
| Behavioral | 3 | 0.1 |
| Biliary Disease - Other | 16 | 0.51 |
| Bone Marrow Disease - Other | 5 | 0.16 |
| Bone Marrow Disease Undetermined | 4 | 0.13 |
| Bronchitis | 10 | 0.32 |
| Cardiac Disease | 161 | 5.18 |
| Chylothorax | 26 | 0.84 |
| Congenital | 44 | 1.42 |
| Cutaneous/Subcutaneous - Other | 10 | 0.32 |
| Diabetes Mellitus | 42 | 1.35 |
| Drug Reaction | 11 | 0.35 |
| FIP | 209 | 6.72 |
| Foreign body | 22 | 0.71 |
| Gastrointestinal Disease - Lower | 6 | 0.19 |
| Gastrointestinal Disease Upper | 65 | 2.09 |
| Hemorrhage - Unknown Cause | 19 | 0.61 |
| Hepatic Lipidosis | 62 | 1.99 |
| Hyperthyroidism | 12 | 0.39 |
| Infection - Bacterial | 36 | 1.16 |
| Infection - Fungal | 28 | 0.9 |
| Infection - Unknown | 5 | 0.16 |
| Infectious - Bacterial | 37 | 1.22 |
| Infectious - Parasitic | 11 | 0.35 |
| Infectious - Viral | 34 | 1.09 |
| LUTD | 26 | 0.84 |
| Meningitis/Meningoencephalitis/Encephalitis | 39 | 1.25 |
| Muscular - Other | 11 | 0.35 |
| Neoplasia | 1,111 | 35.71 |
| Neurologic - Spinal Cord Disease | 16 | 0.51 |
| Neurologic Disease - Other | 3 | 0.1 |
| Neurologic Disease - Unknown | 107 | 3.44 |
| Non-neoplastic Liver Disease | 26 | 0.84 |
| Nutritional | 1 | 0.03 |
| Ophthalmic Disease | 2 | 0.06 |
| Osteoarthritis | 1 | 0.03 |
| Pancreatitis | 35 | 1.13 |
| Peritonitis | 24 | 0.77 |
| Pneumonia | 55 | 1.77 |
| Pyometra | 3 | 0.1 |
| Pyothorax | 23 | 0.74 |
| Renal Failure | 336 | 10.81 |
| Respiratory - Undetermined | 10 | 0.32 |
| Respiratory Lower - Other | 19 | 0.61 |
| Respiratory Upper - Other | 9 | 0.29 |
| Stomatitis | 9 | 0.29 |
| Surgical complication | 9 | 0.29 |
| Thrombus | 100 | 3.22 |
| Toxicity | 13 | 0.42 |
| Trauma | 120 | 3.86 |
| Undetermined | 54 | 1.74 |
| Ureteral obstruction | 15 | 0.48 |
| Vascular | 6 | 0.19 |
