# Supplementary material for: Longevity and mortality in cats: A single institution necropsy study of 3108 cases (1989–2019)
Source: PLoS One. 2022 Dec 29;17(12):e0278199. doi: 10.1371/journal.pone.0278199 (PMC9799304; doi:10.1371/journal.pone.0278199)
Supplement: Supplementary file 3 [file pone.0278199.s002.docx]

Breed info - 6_7_2021 TICA - source of breeds

Breed Classification	Number	Percent	Number with age at time of death	Median Age (years)	IQR (years)	Range (years)
Abyssinian	34	1.09	32	7.14	3.43 - 11.05	0.17 - 20.12
American Shorthair	7	0.23	7	12.44	6.02 - 17.76	4.46 - 18.07
Balinese	3	0.1	2	9.64	8.47 - 10.81	8.47 - 10.81
Bengal	13	0.42	13	1.2	0.77 - 5.03	0.12 - 16.22
Birman	9	0.29	6	4.07	0.76 - 6.84	0.76 - 13.66
Bombay	1	0.03	1	3.24	N/A	N/A
Burmese	30	0.97	30	6.83	1.14 - 11.97	0.01 - 18.96
Chartreux	3	0.1	3	0.69	0.37 - 2.08	0.37 - 2.08
Cornish Rex	1	0.03	1	0.79	N/A	N/A
Cymric	1	0.03	1	6.8	N/A	N/A
Himalayan	52	1.67	49	8.23	4.81 - 11.68	0.19 - 17.36
Japanese Bobtail	1	0.03	1	0.69	N/A	N/A
Maine Coon	53	1.71	52	9.61	4.6 - 13.63	0.34 - 17.29
Manx	28	0.9	27	8.99	4.63 - 13.47	0.54 - 16.77
Mixed breed	2,618	84.23	2501	9.27	4.43 - 13.01	0.01 - 21.85
Norwegian Forest	4	0.13	4	11.24	9.53 - 12.08	8.59 - 12.16
Ocicat	1	0.03	1	0.59	N/A	N/A
Persian	67	2.16	66	6.61	1.5 - 10.32	0.14 - 18.94
Ragdoll	11	0.35	11	6.92	2.47 - 11.64	0.53 - 12.90
Savannah	1	0.03	1	4.07	N/A	N/A
Scottish Fold	8	0.26	8	3.23	0.52 - 11.09	0.33 - 12.82
Siamese	132	4.25	128	9.13	4.38 - 13.34	0.06 - 20.66
Siberian	1	0.03	1	0.58	N/A	N/A
Somali	1	0.03	1	16.69	N/A	N/A
